# Phosphoproteomic dysregulation drives tumor proliferation in Cushing’s disease

**DOI:** 10.1101/2025.09.28.679056

**Published:** 2026-07-28

**Authors:** David T. Asuzu, Dhruval Bhatt, Dustin Mullaney, Debjani Mandal, Diana Nwokoye, Sheelu Varghese, Daniela Tortoza Lopez, Nikhil Ramavenkat, Kory Johnson, Abdel Elkahloun, Zied Abdullaev, Kenneth Aldape, Dragan Maric, Clarisse Quignon, Nasir S. Malik, Joseph P. Steiner, Yan Li, Susan Wray, Christina Tatsi, Lynnette K Nieman, Prashant Chittiboina

## Abstract

Pituitary adenomas constitute up to 20% of primary brain tumors, yet somatic mutations are only found in 15% of pituitary adenomas. Epigenomic dysregulation has been proposed as a tumorigenic mechanism in pituitary adenomas causing Cushing’s disease (CD). We created paired datasets of human CD adenomas and en-route margin adult human pituitary glands and assayed their chromatin accessibility, DNA methylation, transcriptomic, proteomic and phospho-proteomic landscapes. In CD adenomas, we found epigenetic reactivation of a neurodevelopmental phosphoprotein program typically lost in the post-natal pituitary gland. CD cells overexpressed *PPP1R17* , a potent endogenous inhibitor of the ubiquitous protein phosphatase PP2A. Mechanistically, *PPP1R17* overexpression in normal murine pituitary cells recapitulated the adenoma phenotype, and PPP1R17-mediated tumorigenesis was reversible using an FDA-approved small molecule PP2A agonist both in-vitro and in-vivo. Our findings highlight aberrant peptide phosphorylation as a targetable mechanism in CD.

**Significance statement:**

Cushing’s disease (CD) causes significant morbidity and mortality despite best medical and surgical treatment. Surgery is the mainstay of treatment, but carries perioperative risks and is frequently followed by remission. There is a paucity of effective medical treatments, due in part to a limited understanding of tumor mechanisms. The majority of CD adenomas are wild-type, with no known causal mutations. Our study identifies phosphoproteomic dysregulation as a mechanism of CD tumorigenesis common to wild-type and mutant CD adenomas. We target this pathway in-vivo and in-vitro using an FDA-approved small molecule. Our study proposes a novel therapeutic strategy for patients with CD.

## Full Text Availability

The license terms selected by the author(s) for this preprint version do not permit archiving in PMC. The full text is available from the preprint server.

